# Rheological Properties of Konjac Glucomannan Gels and Their Potential Application in Periodontal Therapy

**DOI:** 10.3390/gels12040314

**Published:** 2026-04-07

**Authors:** Annisa Nurrahma Alwiyansyah, Valencia Audrey Halim, Dimas Ilham Hutomo, Yuniarti Soeroso, Benso Sulijaya, Herlis Rahdewati, Nadhia Anindhita Harsas, Robert Lessang, Koichi Tabeta, Fatimah Maria Tadjoedin

**Affiliations:** 1 Periodontology Specialist Program, Department of Periodontology, Faculty of Dentistry, Universitas Indonesia, Jakarta 10430, Indonesia; annisa.nurrahma31@ui.ac.id (A.N.A.); valencia.audrey31@ui.ac.id (V.A.H.); 2 Department of Periodontology, Faculty of Dentistry, Universitas Indonesia, Jakarta 10430, Indonesia; dimas.hutomo@ui.ac.id (D.I.H.); yuniarti@ui.ac.id (Y.S.); benso.sulijaya87@ui.ac.id (B.S.); herlis.rahdewati02@ui.ac.id (H.R.); nadhia.anindhita02@ui.ac.id (N.A.H.); robert_l@ui.ac.id (R.L.); 3 Advancements in Regenerative and Reconstructive Techniques for Periodontal Tissue and Dental Implants Research Group, Faculty of Dentistry, Universitas Indonesia, Salemba Raya No. 4, Jakarta Pusat 10430, Indonesia; 4 Basic, Translational, and Clinical Research in Periodontology Group, Faculty of Dentistry, Universitas Indonesia, Salemba Raya No. 4, Jakarta Pusat 10430, Indonesia; 5 Division of Periodontology, Faculty of Dentistry & Graduate School of Medical and Dental Sciences, Niigata University, Niigata 951-8514, Japan; koichi@dent.niigata-u.ac.jp

**Keywords:** konjac glucomannan, injectable gels, rheological properties, local drug delivery, antibacterial activity, periodontal biomaterials, viscoelastic properties, dentistry

## Abstract

Konjac glucomannan (KGM) is a naturally derived polysaccharide known for its biocompatibility and gel-forming ability and has gained increasing attention in biomaterial and drug delivery research. However, the rheological behavior of KGM gels at clinically relevant concentrations for periodontal use has not been thoroughly investigated. In this study, KGM gels at 0.8%, 1.0%, and 1.2% (*w*/*v*) were prepared and evaluated using oscillatory and steady shear rheology. Rheological analysis revealed increased viscoelastic strength with increasing polymer content, with the 1.2% formulation showing the highest storage modulus, viscosity, and shear stress values across strain, frequency, and temperature ranges. All formulations demonstrated pronounced shear-thinning behavior and dominant elastic characteristics (G′ > G″), indicating stable gel network formation and favorable injectability. The viscoelastic profile remained stable near physiological temperature (37 °C), implying that the gel network can preserve mechanical integrity under intraoral conditions. Gamma irradiation at 15 kGy effectively achieved sterility without visible macroscopic instability, although a qualitative reduction in viscosity was observed. Collectively, these findings indicate that increasing KGM concentration improves mechanical robustness and viscoelastic stability, with the 1.2% gel demonstrating the most favorable rheological profile for potential localized periodontal application.

## 1. Introduction

Periodontitis is a chronic inflammatory disease characterized by progressive destruction of the supporting tissues of the teeth and is primarily associated with dysbiotic biofilms dominated by keystone pathogens such as Porphyromonas gingivalis [1]. Conventional treatment relies on mechanical debridement through scaling and root planing, frequently combined with adjunctive local antimicrobial agents. Although local antibiotic systems, including minocycline-based formulations, have demonstrated clinical effectiveness, concerns regarding antimicrobial resistance and long-term drug exposure continue to drive the search for alternative bio-based materials for localized periodontal therapy [2].

Injectable gel systems have attracted attention in periodontal treatment due to their ability to conform to the periodontal pocket and provide localized retention under dynamic oral conditions [3]. In this context, the rheological properties of gel matrices play a critical role in determining injectability, structural stability, and mechanical performance. Shear-thinning behavior, viscoelastic stability, and network integrity are particularly important for ensuring adequate clinical handling and in situ retention [4].

Konjac glucomannan (KGM) is a naturally derived polysaccharide obtained from *Amorphophallus konjac* tubers and is known for its biocompatibility and gel-forming capability [5]. In terms of its chemical structure, KGM is a predominantly linear polymer composed of D-glucose (G) and D-mannose (M) units linked by β-(1 → 4) glycosidic bonds [6]. Numerous studies have reported that the molar ratio of mannose to glucose is approximately 1.6:1, although this may vary depending on the source of the material [7,8]. The repeating unit of the polymer can be represented by the molecular formula (C_6_H_10_O_5_)_n_. Additionally, a small degree of acetyl substitution also contributes to its solubility and gel-forming capacity [9]. These structural features underlie the distinctive physicochemical and rheological properties of KGM reported in previous studies.

Owing to its high molecular weight and ability to establish physical intermolecular interactions through hydrogen bonding and chain entanglement, KGM can form viscoelastic gel networks with tunable mechanical properties. Previous studies have reported antioxidant, anti-inflammatory, and antibacterial activities of KGM, including inhibitory effects against *P. gingivalis* in systemic models. However, detailed evaluation of the rheological properties of KGM gels at concentrations relevant for localized periodontal applications remains limited [7,10]. Previous study has reported that KGM gels within the concentration of 0.8%, 1.0%, and 1.2% are capable of forming stable viscoelastic gel networks with measurable mechanical properties [11]. These defined concentrations provide a rationale for investigating the rheological behavior of KGM gels for injectable intraoral application.

Therefore, the present study aimed to evaluate the rheological behavior of konjac glucomannan (KGM) gels prepared at selected concentrations to assess their suitability as an injectable gel for potential periodontal applications. In addition, microbiological safety following gamma sterilization were assessed. The findings provide insight into the structure–property relationships of KGM gels and their potential as bio-based gel systems for periodontal applications.

## 2. Results and Discussion

### 2.1. Physical Appearance and Handling Properties

Konjac glucomannan (KGM) gels at concentrations of 0.8%, 1.0%, and 1.2% (*w*/*v*) were successfully prepared using the described formulation method. All formulations appeared homogeneous, translucent, and free of visible particulates, indicating uniform polymer dispersion and proper gel formation. When loaded into plastic syringes, the gels exhibited smooth extrusion behavior without phase separation, demonstrating suitable injectability for localized periodontal application. Following gamma irradiation at 15 kGy, no visible discoloration, precipitation, or phase instability was observed in any formulation. The gels remained visually clear and structurally intact immediately after sterilization, suggesting that the applied sterilization dose did not induce macroscopic degradation of the polymer network. A slight reduction in apparent viscosity during extrusion was noted in the lower concentration formulation, which may reflect partial chain scission or temporary structural rearrangement under mechanical stress. The observed physical stability supports the suitability of the sterilized gels for subsequent rheological and antibacterial evaluation.

### 2.2. Rheological Properties

#### 2.2.1. Strain Sweep Analysis (LVR Determination)

**Figure 1:**
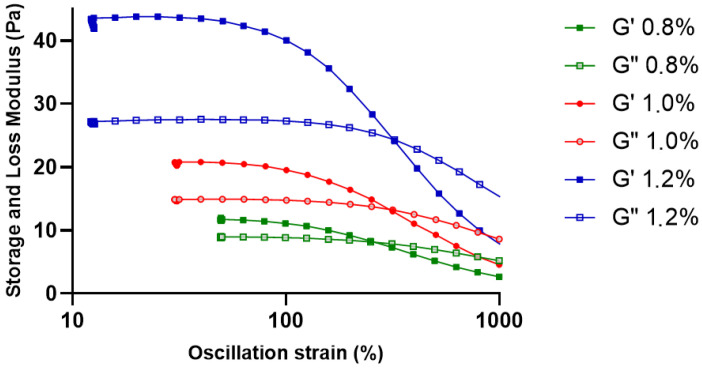
Strain sweep analysis of KGM gels showing the linear viscoelastic region (LVR). Storage modulus (G′) and loss modulus (G″) of KGM gels at concentrations of 0.8%, 1.0%, and 1.2% (*w*/*v*) are presented as a function of oscillatory strain (10–1000%). Solid symbols represent G′ and open symbols represent G″.

Strain sweep analysis was used to identify the linear viscoelastic region (LVR) of the KGM gels in order to better assess the mechanical stability of the gel network (Figure 1). Both G′ and G″ stayed constant at low strain amplitudes, suggesting that the internal gel structure was maintained during slight deformations. G′ gradually decreased as strain increased, indicating the beginning of structural breakdown in the polymer network [12]. The gels appear to maintain mostly elastic properties even with increasing distortion, as seen by the lack of a distinct crossover point between G′ and G″ throughout the studied strain range [12,13].

The 1.2% KGM gel consistently exhibited the highest modulus values and the widest viscoelastic stability range among the tested formulations, with the 1.0% and 0.8% gels coming in second and third. This pattern suggests that the three-dimensional gel network is strengthened and the density of intermolecular contacts is increased with increasing polymer concentration [14]. These findings demonstrate that increasing polymer concentration improves structural robustness and deformation resistance of KGM gels, which are important characteristics for maintaining mechanical integrity under dynamic mechanical stresses [15]. Based on these results, a strain value within the LVR (1%) was selected for subsequent oscillatory measurements.

#### 2.2.2. Temperature Sweep Analysis

**Figure 2:**
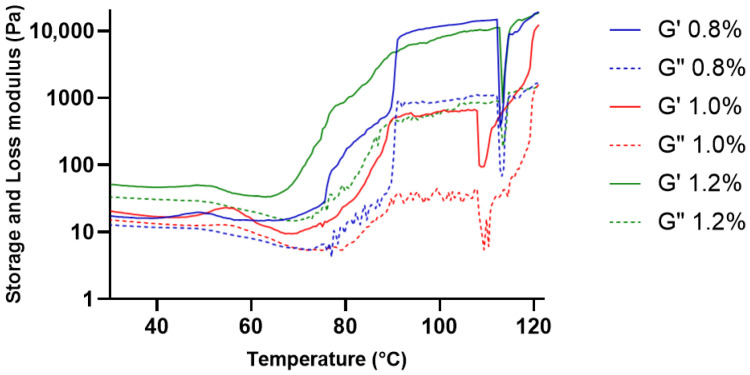
Viscoelastic response of KGM gels during heating. Storage modulus (G′) and loss modulus (G″) of KGM gels at concentrations of 0.8%, 1.0%, and 1.2% (*w*/*v*) measured over the temperature range of 30–120 °C. Solid lines represent G′ and dashed lines represent G″.

The viscoelastic response of KGM gels during heating was evaluated over the temperature range of 30–120 °C (Figure 2). At temperatures between 30 and 60 °C, both the storage modulus (G′) and loss modulus (G″) demonstrated consistently low and stable values across all concentrations, indicating a structured yet moderately flexible gel network under mild thermal conditions. Throughout this temperature range, G′ constantly exceeded G″, confirming the dominance of elastic behavior [13]. However, a decrease in modulus was observed at temperatures above approximately 100 °C.

The observed viscoelastic characteristics are typical of physically crosslinked polysaccharide gels, where the network structure is stabilized by intermolecular interactions such as hydrogen bonding and chain entanglement [16]. The elastic dominance (G′ > G″) in this temperature range suggests the formation of a continuous viscoelastic network within the gel matrix. This structural organization enables the gel matrix to maintain mechanical coherence under mild thermal conditions, which is an important characteristic for biomedical gel systems intended to preserve their structural integrity during handling and application [17].

#### 2.2.3. Oscillation Rheological Behavior

The viscoelastic stability of the KGM gels under small deformation conditions was further assessed through oscillatory rheological tests. These analyses included temperature-dependent oscillatory measurements within the physiologically temperature range as well as strain sweep analysis to determine the linear viscoelastic region (LVR) of the gel network. The thermal stability of the viscoelastic structure is illustrated by temperature sweep analysis, whereas the mechanical stability of the gel network is defined by strain sweep analysis as deformation increases [18].

**Figure 3:**
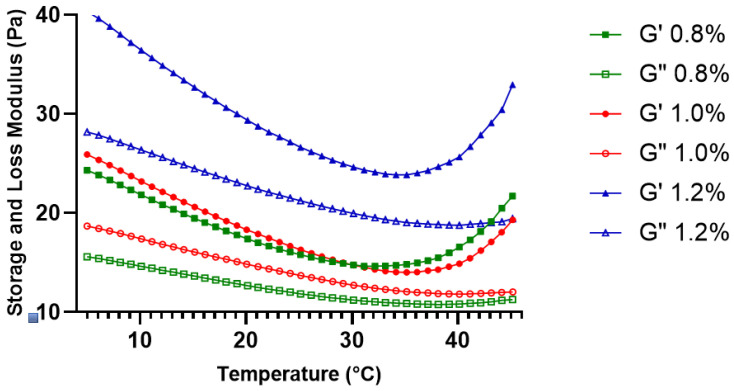
Temperature-dependent viscoelastic behavior of KGM gels (0.8%, 1.0%, and 1.2% *w*/*v*) evaluated by oscillatory temperature sweep (5–45 °C). Solid symbols represent G′ and open symbols represent G″.

Figure 3 illustrates that both the storage modulus (G′) and loss modulus (G″) progressively diminished as the temperature rose from 5 °C to about 35–40 °C across all evaluated concentrations. This reduction in modulus values can be attributed to increased polymer chain mobility and partial weakening of intermolecular interactions as thermal energy increases [19].

This finding indicates that the gel matrix undergoes reversible structural adaptation rather than irreversible thermal degradation within this temperature range. Throughout the entire temperature sweep, G′ remained consistently higher than G″ for all concentrations, confirming the dominance of elastic behavior and the absence of a gel–sol transition [20]. Based on these findings, the temperature sweep results indicate that KGM gels maintain structural integrity within the range relevant to intraoral conditions, with concentration-dependent modulation of thermal mechanical stability.

#### 2.2.4. Steady Shear Flow Behavior

**Figure 4:**
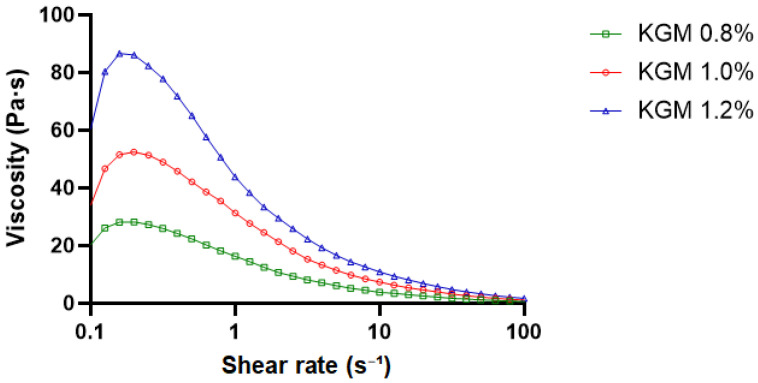
Steady shear viscosity profiles of KGM gels at concentrations of 0.8%, 1.0%, and 1.2% (*w*/*v*). Apparent viscosity as a function of shear rate (0.1–100 s^−1^) measured at 25 °C. All formulations exhibited shear-thinning behavior, with viscosity increasing as polymer concentration increased.

The steady shear viscosity profiles of KGM gels at concentrations of 0.8%, 1.0%, and 1.2% are presented in Figure 4. All formulations exhibited a pronounced decrease in apparent viscosity with increasing shear rate (0.1–100 s^−1^), indicating typical shear-thinning (pseudoplastic) behavior [21,22]. At low shear rates (≤1 s^−1^), the apparent viscosity was highest for the 1.2% formulation (approximately 75–85 Pa·s), followed by 1.0% (≈45–50 Pa·s) and 0.8% (≈25–30 Pa·s). The higher zero-shear viscosity observed at increased concentrations reflects enhanced intermolecular interactions and chain entanglement density within the gel network. As shear rates increased, viscosity values of all concentrations converged toward lower levels, suggesting structural rearrangement and partial disruption of the gel network under applied shear [23,24,25].

#### 2.2.5. Dynamic Frequency Sweep Analysis

**Figure 5:**
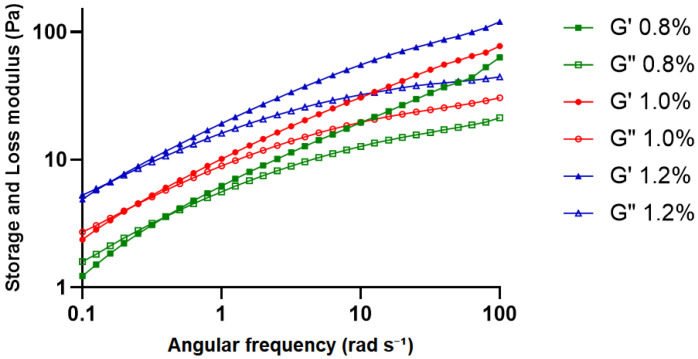
Dynamic frequency sweep of KGM gels at concentrations of 0.8%, 1.0%, and 1.2% (*w*/*v*). Storage modulus (G′) and loss modulus (G″) are shown as a function of angular frequency (0.1–100 rad s^−1^) measured at 25 °C and 1% strain (within the linear viscoelastic region). Solid symbols represent G′ and open symbols represent G″.

The dynamic frequency sweep analysis of KGM gels was performed within the linear viscoelastic region (1% strain) over the angular frequency range of 0.1–100 rad s^−1^ (Figure 5). For all concentrations, both storage modulus (G′) and loss modulus (G″) increased progressively with increasing frequency, while G′ remained consistently higher than G″ throughout the tested range. This behavior indicated that gels retained elastic-dominant viscoelastic characteristics over the applied frequency range.

The magnitude of both moduli increased with polymer concentration, with the 1.2% KGM gel showing the highest values, followed by the 1.0% and 0.8% formulations. This concentration-dependent increase in modulus reflects a denser and more cohesive viscoelastic network, in which polymer chains are more effectively associated through hydrogen bonding and chain entanglement. The relatively stable separation between G′ and G″ across the frequency range further supports the formation of a structured gel network capable of maintaining mechanical integrity under oscillatory deformation [26,27].

These rheological characteristics are also particularly relevant for injectable periodontal systems. Previous study have emphasized that biomaterial gels intended for periodontal applications should exhibit shear-thinning flow behavior and viscoelastic stability, enabling ease of injection while maintaining structural integrity and retention within the periodontal pocket [28]. Intraoral temperature has been reported to most frequently range between 35 and 36 °C over 24 h, with typical fluctuations within 33–37 °C. All formulations maintained elastic dominance within the physiologically relevant temperature range, indicating structural stability under intraoral conditions [18].

Although the present rheological characterization provides comprehensive qualitative insight into the concentration-dependent viscoelastic behavior and shear-responsive flow properties of KGM gels, further quantitative analysis would be valuable to deepen mechanistic understanding. In particular, future studies may incorporate constitutive rheological modeling approaches, statistical evaluation of rheological parameters, and time-dependent structural recovery assessments to enable more thorough comparison with other injectable hydrogel systems.

### 2.3. ATR-FTIR Analysis of KGM Gel

**Figure 6:**
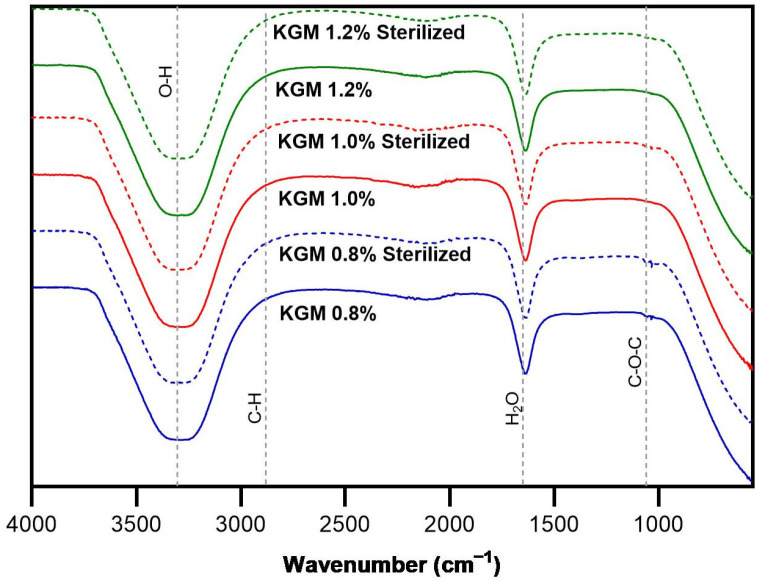
ATR-FTIR spectra of konjac glucomannan (KGM) gels at concentrations of 0.8%, 1.0%, and 1.2% (*w*/*v*) before and after gamma irradiation sterilization (15 kGy).

ATR-FTIR spectroscopy was performed to evaluate potential structural changes in KGM gels before and after gamma sterilization. Similar distinctive absorption bands indicative of konjac glucomannan were seen in the spectra of the sterilized and non-sterilized samples (Figure 6). In transmittance spectra, these bands appear as downward peaks. A broad band observed at approximately 3300–3400 cm^−1^ corresponds to O–H stretching vibrations associated with hydroxyl groups involved in hydrogen bonding within the polysaccharide structure. The glucomannan backbone’s C–H stretching vibrations are responsible for the peak about 2920 cm^−1^ [29].

Furthermore, absorption bands at approximately 1640 cm^−1^ are frequently linked to bound water in the hydrophilic polysaccharide matrix. The C–O and C–O–C stretching vibrations of the β-1,4-linked glucomannan chains are represented by strong peaks in the 1000–1100 cm^−1^ range [30].

Comparison of the spectra before and after gamma irradiation did not reveal the appearance of new peaks or significant shifts in the characteristic bands. This implies that after sterilization, the KGM polysaccharide backbone’s core chemical structure was mostly retained. The findings show that while there may be slight physical alterations in the gel network, the applied irradiation dose (15 kGy) did not cause significant chemical degradation of the KGM matrix.

These findings highlight the usefulness of ATR-FTIR analysis for evaluating the structural integrity of KGM gels and provide a useful study for future research at elucidating structural and chemical changes during gel formation and processing.

### 2.4. Sterilization and Microbiological Evaluation

Since materials intended for biomedical and intraoral applications must be sterile prior to clinical use, the feasibility of sterilizing the KGM gels using gamma irradiation was evaluated. Sterilization was performed using gamma irradiation at a dose of 15 kGy. The selected dose was based on the principles outlined in ISO 11137-2 for radiation sterilization of health care products [31]. The selected dose was intended to ensure effective microbial reduction while minimizing potential alterations to the physicochemical and rheological properties of the KGM gel matrix.

**Figure 7:**
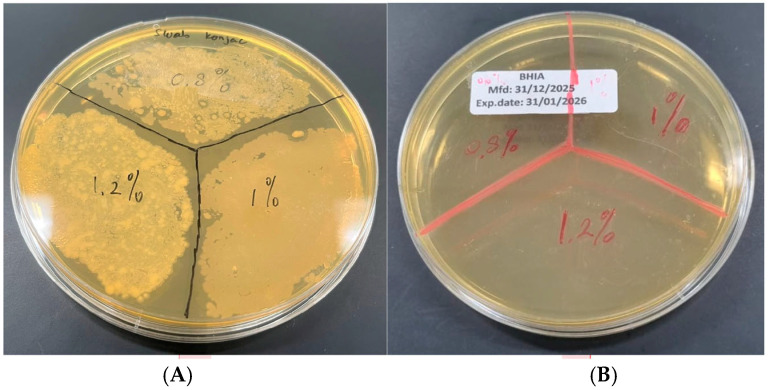
Microbial evaluation of KGM gels following sterilization. Representative BHI agar plates inoculated by surface swabbing of KGM gels at concentrations of 0.8%, 1.0%, and 1.2% (*w*/*v*): (**A**) non-sterilized samples and (**B**) gamma-sterilized samples. Extensive bacterial colony formation was observed in non-sterilized gels, whereas no visible microbial growth was detected in sterilized samples.

Microbial evaluation was performed by surface swabbing of KGM gels followed by inoculation onto Brain Heart Infusion (BHI) agar plates. Representative results are shown in Figure 7. The non-sterilized KGM gels (0.8%, 1.0%, and 1.2% *w*/*v*) exhibited extensive bacterial growth after incubation, characterized by dense and heterogeneous colony formation across all concentrations. In contrast, no visible bacterial colonies were observed on plates inoculated from gamma-sterilized KGM gels, indicating effective elimination of microbial contamination. The absence of growth across all tested concentrations demonstrates that gamma irradiation at 15 kGy was sufficient to achieve sterility under the applied conditions.

These results confirm that gamma irradiation provides an effective sterilization strategy for KGM gels intended for biomedical use. The sterilization process did not induce visible macroscopic changes in gel appearance, suggesting preservation of structural integrity following irradiation. Although no visible macroscopic changes such as discoloration or phase separation were observed after sterilization, a qualitative reduction in viscosity was noted during manual extrusion from the syringe. The sterilized gels appeared slightly less resistant to flow compared to non-sterilized samples. However, no post-irradiation rheological measurements were performed to quantitatively assess this change.

## 3. Conclusions

KGM gels at concentrations of 0.8%, 1.0%, and 1.2% were successfully formulated and exhibited concentration-dependent viscoelastic behavior. Oscillatory and steady shear analyses demonstrated that increasing polymer concentration enhanced storage modulus, shear stress, and viscosity, while maintaining predominant elastic, gel-like characteristics across the evaluated deformation ranges. All formulations showed pseudoplastic flow behavior, which is advantageous for injectable applications requiring controlled flow under shear.

Gamma irradiation at 15 kGy effectively achieved sterilization without inducing visible macroscopic instability, although a qualitative decrease in viscosity was observed following sterilization. The gels retained structural integrity and functional consistency within the tested concentration range.

Taken together, the combination of shear-responsive flow behavior and elastic network dominance may support local retention and handling performance during clinical placement in the context of periodontal therapy. These findings support the potential applicability of konjac glucomannan gels as injectable biomaterials for localized periodontal therapy, while highlighting the importance of further quantitative rheological optimization and sterilization-related structural evaluation to enhance their clinical performance and functional reliability.

Future research should evaluate the antimicrobial activity of KGM gels against periodontal pathogens and further explore their role in wound healing and periodontal tissue regeneration following inflammation through in vitro studies, followed by in vivo studies and clinical investigations to confirm their therapeutic potential. We hope these KGM gels can offer a more affordable and sustainable alternative to conventional antibiotic therapies, potentially reducing antimicrobial resistance while expanding the treatment options beyond current gold-standard approaches in periodontal infection management.

## 4. Materials and Methods

### 4.1. Materials

Konjac glucomannan (KGM) powder (CAS No. 37220-17-0) was purchased from Chengdu Root Industry Co., Ltd. (Chengdu, China). According to the supplier’s certificate of analysis, the material contained approximately 94% glucomannan, with a white appearance, viscosity of 37,000 mPa·s, particle size of 120–200 mesh, ash content of 1.8%, and moisture content of 9.2%. Sodium carbonate (Na_2_CO_3_, analytical grade) was obtained from the Chemistry Laboratory, Faculty of Mathematics and Natural Sciences, Universitas Indonesia (Depok, Indonesia). Brain Heart Infusion (BHI) agar was prepared and supplied by the Oral Biology Laboratory, Faculty of Dentistry, Universitas Indonesia (Depok, Indonesia). Distilled water was used for all preparations. All materials were used as received without further purification.

### 4.2. Preparation of KGM Gel

KGM gels were prepared at concentrations of 0.8%, 1.0%, and 1.2% (*w*/*v*). A sodium carbonate solution was first prepared by dissolving 0.1 g of Na_2_CO_3_ in 500 mL of distilled water under manual agitation until completely dissolved. Subsequently, 100 mL of this solution was used as the dispersing medium for gel preparation.

KGM powder (0.4 g, 0.5 g, and 0.6 g for 0.8%, 1.0%, and 1.2% respectively) was gradually wetted and dispersed into the Na_2_CO_3_ solution in small portions to prevent agglomeration. The mixture was stirred at 400 rpm at 40 °C until a homogeneous dispersion was obtained, followed by heating at 90 °C for 1 h to induce gel formation. No chemical crosslinker was used in this formulation. The gel preparation procedure was adapted from the method reported by Tong et al. with slight modifications [11]. Gel formation occurred through alkali-induced heat set gelation under alkaline conditions and thermal treatment. The gels were allowed to cool to room temperature and stored at 4 °C before further analysis.

For sterilization studies, the gels were transferred into sterile 1 mL syringes, sealed, and subjected to gamma irradiation at a dose of 15 kGy (Rel-Ion Sterilization Services, Bekasi, Indonesia), following radiation sterilization guidelines described in ISO 11137-2 [31].

### 4.3. Rheological Measurements

Rheological measurements were performed at the ILRC Laboratory, Universitas Indonesia (Depok, Indonesia), using a Dynamic Hybrid Rheometer (DHR, TA Instruments, New Castle, DE, USA) equipped with a parallel plate geometry (stainless steel, 20 mm diameter). The gap between plates was adjusted according to instrument recommendations at 1.0 mm to ensure uniform gel distribution during measurements.

Approximately 1 mL of each gel sample was carefully placed onto the lower plate, and the upper plate was lowered until the predefined gap was reached. All measurements were conducted at a controlled temperature of 25 °C unless otherwise specified, using the instrument’s integrated temperature control system.

Within the linear viscoelastic region (LVR) identified by strain sweep data, oscillatory rheological studies were carried out. While oscillatory temperature sweep measurements were carried out between 5 and 45 °C under physiologically appropriate conditions, temperature sweep measurements were carried out between 30 and 120 °C. Dynamic frequency sweep measurements were performed at a constant strain amplitude of 1% over an angular frequency range of 0.1–100 rad s^−1^.

To assess the viscosity and shear stress responses of the gels, steady shear measurements were carried out using shear rates ranging from 0.1 to 100 s^−1^. Unless otherwise noted, the instrument’s integrated temperature control system was used to perform all measurements at 25 °C.

### 4.4. ATR-FTIR Analysis

Potential structural alterations in KGM gels before and after gamma sterilization were examined using Fourier transform infrared spectroscopy with attenuated total reflectance (ATR-FTIR) at ILRC Laboratory, Universitas Indonesia (Depok, Indonesia). An FTIR spectrometer (Thermo Fisher Scientific, Waltham, MA, USA) with an ATR attachment was used to record the spectra. 16 scans were obtained for every sample, and measurements were made over a wavenumber range of 4000–500 cm^−1^ with a spectral resolution of 4 cm^−1^. To find potential alterations in distinctive functional group vibrations, the spectra of gamma-irradiated and non-sterilized KGM gels were compared.

### 4.5. Gel Sterilization

Sterility testing was conducted to evaluate microbial contamination in both sterilized and non-sterilized KGM gel formulations. Surface swabbing was performed using sterile swabs, which were subsequently inoculated onto Brain Heart Infusion (BHI) agar plates. The inoculated plates were incubated at 37 °C for 24 h. After incubation, plates were visually examined for the presence or absence of microbial growth.

### 4.6. Flowchart

**Figure 8:**
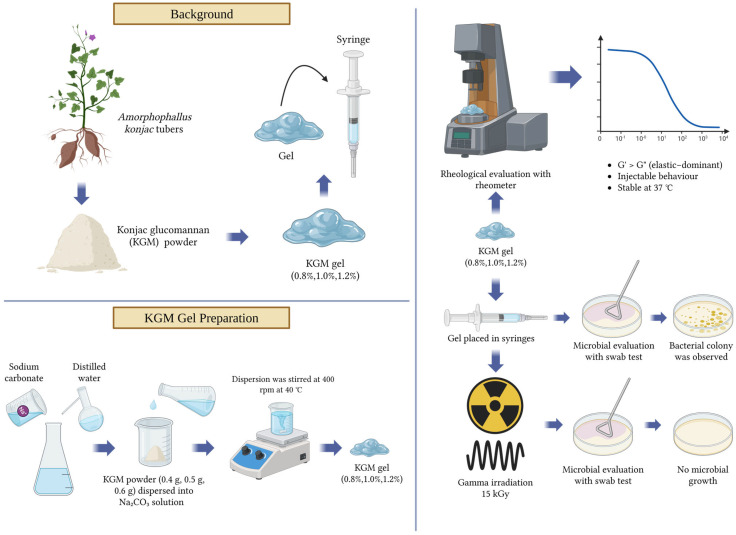
Illustration of overall workflow and methodology (Created in Biorender https://BioRender.com).

The overall workflow of this study, including KGM gel preparation, sterilization, rheological characterization, and biological evaluation, is illustrated in Figure 8.

## Data Availability

The data presented in this study are available upon request from the corresponding author.
